# Ethical and Regulatory Considerations in the Clinical Translation of Pluripotent Stem Cell‐Derived NK Cell Therapies

**DOI:** 10.1111/cpr.70129

**Published:** 2025-09-29

**Authors:** Qianwen Chen, Jianwei Lv, Xinwei Xie, Hanlin Zhu, Zhenyu Xiao, Yaojin Peng

**Affiliations:** ^1^ State Key Laboratory of Organ Regeneration and Reconstruction Institute of Zoology, Chinese Academy of Sciences Beijing China; ^2^ Beijing Institute for Stem Cell and Regenerative Medicine Beijing China; ^3^ School of Life Sciences Xiamen University Xiamen China; ^4^ School of Life Science Beijing Institute of Technology Beijing China; ^5^ University of the Chinese Academy of Sciences Beijing China

**Keywords:** ethics, governance, natural killer cells, pluripotent stem cells, regulation

## Abstract

Advancements in the generation of human pluripotent stem cell‐derived natural killer (PSC‐NK) cells have attracted considerable attention within the biomedical research community, offering a promising off‐the‐shelf technique for universal immune therapy. However, this technique is associated with certain ethical, safety, and regulatory challenges, including ensuring genomic stability, preventing contamination and adhering to rigorous ethical standards for cell sourcing and obtaining informed consent. Addressing these challenges would require robust quality control, transparent data‐sharing practices, and cross‐border collaboration to ensure alignment with ethical and scientific standards. Future development must therefore focus on patient safety, data privacy and equitable access within a comprehensive ethical framework. These measures are crucial for maintaining public trust in and enabling the responsible clinical integration of PSC‐NK therapies, thereby supporting their advancement while maintaining a balance between innovation and societal and ethical considerations.

## Graphical abstract

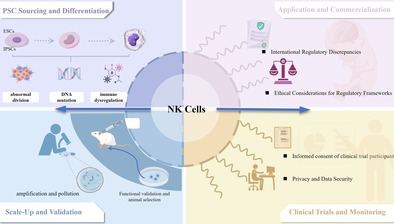

## 1 Introduction

Progress in stem cell research has enabled the differentiation of natural killer (NK) cells from pluripotent stem cells (PSCs), allowing for enhanced genetic engineering compatibility and the production of off‐the‐shelf cell products [1, 2, 3, 4]. PSC‐derived NK (PSC‐NK) cells are expected to emerge as a promising ‘cell drug’ for universal cancer therapy, and they have already been integrated into many clinical trials worldwide [5]. PSC‐NK technology has reshaped fields involving experimental stem cells and immunotherapy, particularly fields that have been the subject of major ethical discussions, such as animal experimentation and the use of human‐derived materials in research [6, 7]. Although many of these ethical concerns are common across stem cell research, the major public attention that PSC‐NK cells have attracted and the expectations associated with their therapeutic potential warrant a dedicated examination [8].

Immunotherapy has been widely applied in the treatment of various cancers, with the FDA approving a range of therapies including immune checkpoint inhibitors and antibody‐drug conjugates [1]. Meanwhile, numerous novel approaches are being explored in ongoing clinical trials. NK cell therapy, which exerts direct cytotoxic effects on tumour cells without requiring prior antigen recognition, offers distinct advantages within the tumour microenvironment [9]. However, the limited availability of NK cells remains a key bottleneck in cellular immunotherapy [10]. PSC‐NK cells not only enable scalable and standardised manufacturing, but also represent a promising off‐the‐shelf platform with broad therapeutic potential [11].

While the ethical and regulatory concerns surrounding PSC‐NK cells echo longstanding issues associated with PSCs—such as cell sourcing, informed consent and genomic stability—the unique attributes of PSC‐NK technologies introduce distinct challenges [3, 7, 12]. Extensive gene editing, including CRISPR‐based modifications, raises the spectre of off‐target effects and immune dysregulation [13]. Immune‐mediated risks, such as graft rejection, malignant transformation and cytokine release syndrome, further underscore the need for rigorous safeguards [14]. Moreover, their dependence on genetic profiling amplifies ethical questions related to privacy and data governance [15]. Anchored in the broader ethical discourse on PSC‐based therapies, this study delineates the specific risk landscape of PSC‐NK technologies and proposes a governance framework to support their responsible research and translation.

## 2 PSC‐NK Technology: Foundations and Challenges

Cancer is a highly complex disease and remains one of the leading causes of mortality worldwide. Despite major advancements having been made in cancer treatment, including with surgical resection, radiation, chemotherapy and targeted therapies involving small molecule inhibitors or monoclonal antibodies, cancer interventions are often associated with major adverse effects. In recent years, immune cell‐based therapies have emerged as a promising strategy to mitigate adverse effects while effectively targeting both hematologic malignancies and solid tumours. NK cells are an integral component of this technique; they play a pivotal role in the initial immune response against malignant cells [16]. Notably, NK cells have been used to treat various types of cancer without a need for stringent human leukocyte antigen matching [17].

Compared to NK cells derived from peripheral blood or umbilical cord blood, PSC‐NK cells offer several advantages that make them an attractive alternative for cancer immunotherapy. Natural NK cells are limited by donor variability, restricted proliferative capacity, and challenges in consistent expansion and genetic modification [9, 18]. In contrast, PSC‐NK cells can be derived from well‐characterised PSC lines, enabling reproducible and large‐scale manufacturing with higher batch‐to‐batch consistency [19, 20]. Furthermore, the pluripotent nature of PSCs facilitates precise genetic engineering prior to differentiation, allowing for the incorporation of features such as chimeric antigen receptors (CARs), immune checkpoint deletions, and resistance to tumour‐induced immunosuppression [21]. These engineered PSC‐NK cells not only exhibit enhanced cytotoxicity and persistence in vivo but also provide a scalable, standardised, and potentially cost‐effective ‘off‐the‐shelf’ immunotherapy product [3]. Thus, PSC‐NK technology represents a promising next‐generation platform that addresses many of the limitations associated with natural NK cell therapies.

### 2.1 Differentiation From PSCs to NK Cells

**FIGURE 1:**
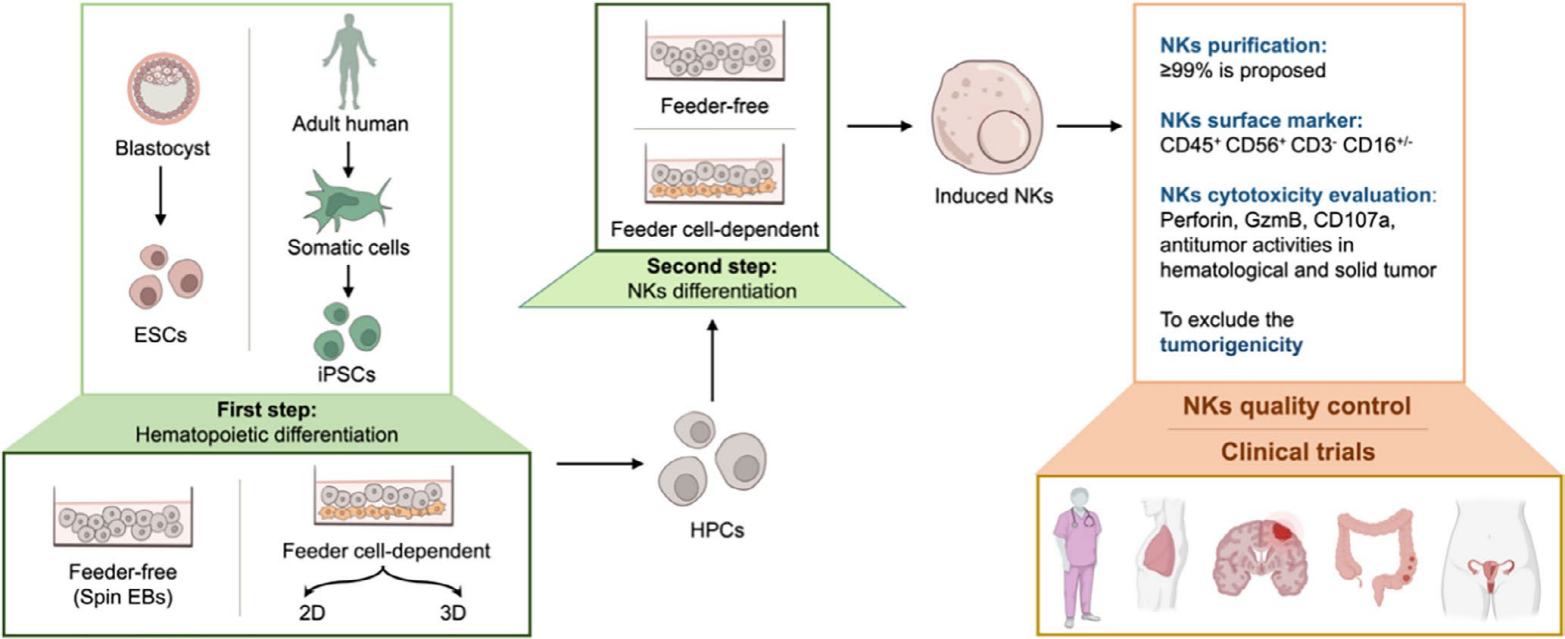
Methods for obtaining NK cells through PSC differentiation.

Human NK cells can be generated in vitro from PSCs, including embryonic stem cells (ESCs), derived from blastocysts, and induced pluripotent stem cells (iPSCs), which are reprogrammed from somatic cells. The differentiation process typically begins with co‐culturing PSCs on irradiated mouse bone marrow stromal cell lines, such as M210‐B4 or S17, for 14–17 days [2], yielding CD34^+^ haematopoietic progenitor cells (HPCs)—early blood cell precursors marked by surface expression of CD34 (see Figure 1). These CD34^+^ HPCs are subsequently isolated and induced to differentiate into NK cells via co‐culture with foetal liver‐derived stromal cells (e.g., AFT024) in the presence of defined cytokines, including stem cell factor, IL‐3, IL‐7, IL‐15 and Flt3L. Over time, this stepwise protocol results in the generation of functionally mature NK cells [3].

Optimised protocols for PSC‐NK cell generation have incorporated spin embryoid body (EB) techniques, in which EBs are transferred into NK differentiation conditions with or without stromal support. Feeder cells such as K562 engineered to express membrane‐bound IL‐21 have been employed to promote robust NK cell expansion [4]. However, the continued reliance on xenogeneic stromal cells poses safety concerns that complicate clinical translation. Moreover, spin EB‐based approaches are constrained by issues of efficiency, batch‐to‐batch variability, and scalability, limiting their suitability for large‐scale clinical manufacturing. To overcome these challenges, Huang et al. [22] developed an organoid‐based, feeder‐free differentiation platform that bypasses the need for embryoid bodies by leveraging lateral plate mesoderm (LPM) cells—embryonic progenitors of the cardiovascular system and coelomic lining [16]. LPM‐derived cells contribute to the endogenous haematopoietic niche by supporting haematopoietic stem cell (HSC) emergence, thereby providing a physiologically relevant microenvironment for efficient NK cell generation at scale [17]. Differentiation of NK cells from both iPSCs and ESCs has been achieved under feeder‐dependent and feeder‐free conditions [23].

Prior to clinical application—such as in cancer immunotherapy—PSC‐NK products undergo rigorous quality control procedures, including purification, immunophenotyping, cytotoxicity assays, and tumourigenicity testing, to ensure both safety and efficacy. Preclinical animal models play a pivotal role in this translational process. In a subcutaneous pancreatic cancer model, treatment with NK cells engineered to target FR/DR4 significantly enhanced tumour infiltration and selective apoptosis of cancer cells [23].

### 2.2 PSC‐NK Engineering and Clinical Evolution

Unlike natural NK cells, which have limited efficiency in genome editing and genetic engineering, PSC‐NK cells offer a highly tractable platform for genetic engineering at the pluripotent stage. These approaches are designed to enhance the growth capacity, in vivo persistence and cytotoxic potential of PSC‐NK cells, thereby increasing their antitumor efficacy [2]. Enhancing the functions of NK cells typically involves the incorporation of specific antigen recognition structures, the modification of certain receptors and the adjustment of pathways regulating NK cell activity.

The anticancer potential of NK cells can be improved through antibody‐dependent cellular cytotoxicity [24]. This mechanism facilitates the recognition and targeting of cancer cells by NK cells. In NK cells, certain internal regulatory mechanisms can be modified to augment their anticancer capabilities. For instance, gene (e.g., CD38) knockouts targeting negative regulators of NK cell activity, as well as the addition of specific immune‐stimulating molecules, have yielded positive functional outcomes [1]. Clustered regularly interspaced short palindromic repeats (CRISPR) technology, which is used to eliminate inhibitory proteins in PSCs, has also yielded NK cells with extended in vivo persistence and enhanced anticancer effects [24].

The clinical translation of genome‐edited PSC‐NK cells has accelerated in recent years, with several candidates advancing into early‐phase clinical trials for both hematologic and solid malignancies. For instance, iPSC‐derived NK products—such as FT500, FT516, FT538, FT576 and FT596—are being evaluated across a spectrum of indications, including lymphoma, leukaemia, multiple myeloma, and advanced solid tumours (see Table 1). These engineered cell therapies frequently incorporate targeted modifications, including checkpoint receptor deletions and CAR constructs, to enhance antitumour cytotoxicity, persistence and specificity. Collectively, these trials underscore the feasibility of scalable, off‐the‐shelf PSC‐NK platforms and represent a pivotal advance towards broader clinical implementation.

**TABLE 1:** Clinical trials of iPSC‐NKs and off‐the‐shelf cellular immunotherapy products.

Clinical trials based on iPSC‐NKs therapy for human disease registered in ClinicalTrials.gov					
First posted date	NKs source	Identifiter	Phase	Disease	Comment
2019/2/15	iPSC‐NKs (FT500)	NCT03841110	I	Solid tumour	FT500, iPSC‐NKs immunotherapy product, used alone or in combination with an immune checkpoint inhibitor (ICI) in Phase I clinical trial for advanced solid tumour. https://clinicaltrials.gov/ct2/show/record/NCT03841110
2019/9/26	iPSC‐NKs (FT500)	NCT04106167	Observational studies	Advanced solid tumour	Evaluation long‐term safety and efficacy for patients who have previously participated in the FT500‐101 study and received allogeneic iPSC‐NKs immunotherapy. https://clinicaltrials.gov/ct2/show/record/NCT04106167
2020/11/16	iPSC‐NKs (FT516)	NCT04630769	I	Recurrent ovarian cancer, fallopian tube, primary peritoneal, cavity cancer	FT516 and IL‐2 combined with Enoblituzumab for ovarian cancer and this was the first intraperitoneal injection of FT516. https://clinicaltrials.gov/ct2/show/record/NCT04630769
2022/7/27	iPSC‐NKs (FT516)	NCT04023071	I	B‐cell lymphoma	A Phase 1/1b chinical trial evaluating the antitumor effect of FT516 monotherapy in relapsed/refractory acute myeloid leukaemia (AML) and combined with CD20 directed monoclonal antibodies in B‐cell lymphoma. https://clinicaltrials.gov/ct2/show/record/NCT04023071
2020/9/16	iPSC‐NKs (FT516)	NCT04551885	I	Advanced solid tumours	A dose‐finding clinical trial of FT‐516 with monoclonal antibodies to treat with advanced solid tumours in Phase I. https://clinicaltrials.gov/ct2/show/record/NCT04551885
2020/4/27	iPSC‐NKs (FT516)	NCT04363346	I	Coronavirus Disease 2019 (COVID‐19)	A Phase I clinical trial to determine the maximum tolerated dose (MTD) of FT516 for the treatment of COVID‐19. https://clinicaltrials.gov/ct2/show/NCT04363346
2022/5/27	iPSC‐NKs (FT536)	NCT05395052	I	Advanced solid tumours	A Phase I dose‐finding study of FT536 monotherapy and in combination with monoclonal antibodies. https://clinicaltrials.gov/ct2/show/record/NCT05395052
2022/8/30	iPSC‐NKs (FT538)	NCT04714372	I	AML	A Phase I clinical trial to identify MTD of FT538 combined with Daratumumab in treating with AML. https://clinicaltrials.gov/ct2/show/NCT04714372
2021/10/6	iPSC‐NKs (FT538)	NCT05069935	I	Advanced solid tumours	A clinical trial of dose‐finding in FT538 combine with monoclonal antibody following lymphodepletion in subjects with advanced solid tumours in Phase I. https://clinicaltrials.gov/ct2/show/record/NCT05069935
2022/1/10	iPSC‐NKs (FT576)	NCT05182073	I	Multiple Myeloma (MM)	A Phase I clinical dose‐finding of FT576 as a monotherapy and in combination with MM. https://clinicaltrials.gov/ct2/show/NCT05182073
2020/9/21	iPSC‐NKs (FT596)	NCT04555811	I	Non‐Hodgkin Lymphoma (NHL)	A Phase I clinical study of FT596 and rituximab in preventing recurrence after autologous haematopoietic stem cell transplantation for NHL. https://clinicaltrials.gov/ct2/show/record/NCT04555811
2020/1/29	iPSC‐NKs (FT596)	NCT04245722	I	Relapsed/Refractory B‐cell Lymphoma and Chronic Lymphocytic Leukaemia	A Phase I clinical dose‐finding of FT596 as monotherapy and in combination with Rituximab or Obinutuzumab in subjects with relapsed/refractory B‐cell Lymphoma or Chronic Lymphocytic Leukaemia. https://clinicaltrials.gov/ct2/show/record/NCT04245722

As summarised in Table 1, PSC‐NK cell therapies have evolved through successive generations of engineering. Early products like FT500 represent minimally modified iPSC‐NK cells used alone or with checkpoint inhibitors [25]. Second‐generation therapies, such as FT516, incorporate membrane‐bound IL‐15 to improve persistence and efficacy [26]. The most advanced candidate, FT596, integrates multiple enhancements including a non‐cleavable, high‐affinity CD16a receptor, a CD19‐specific CAR, and IL‐15 expression—into a single, off‐the‐shelf product for scalable immunotherapy [27]. These innovations improve cytotoxicity, specificity and longevity but also raise ethical and safety concerns, including immune overactivation and the need for long‐term monitoring, necessitating adaptive oversight.

### 2.3 Advantages and Translational Hurdles

PSC‐NK cells present distinct advantages over natural NK cells from peripheral or cord blood. PSC‐NKs exhibit robust cytotoxicity comparable to primary NK cells [19], and their derivation from renewable hPSC lines enables unlimited expansion and precise genetic engineering, facilitating standardised, off‐the‐shelf immunotherapies [28]. Knockout of inhibitory receptors such as NKG2A further enhances their antitumour and antiviral function [28]. In contrast, natural NK cells rapidly lose effector functions and acquire tissue‐resident phenotypes [29], lack antigen‐specific receptors [30], and are vulnerable to immunosenescence [31]. Their functional activity is regulated by a fragile balance of activating and inhibitory signals [30], and isolating highly active NK subsets remains technically demanding [30]. These limitations underscore the superiority of PSC‐NKs as a scalable and programmable platform for next‐generation cancer immunotherapy.

Despite these advantages, PSC‐derived cell therapies—including PSC‐NK cells—face a spectrum of shared challenges across both technological and clinical domains, including issues of cell sourcing and differentiation, genomic stability, immunogenicity and in vivo persistence. First, in terms of cell sourcing and differentiation, all PSC‐based therapies require optimised differentiation protocols to minimise the risk of residual undifferentiated cells. PSC‐NK cells, in particular, often exhibit insufficient maturity, leading to the generation of functionally impaired immature NK cells, which compromises their cytotoxic efficacy [32]. Second, regarding genomic stability, while all PSC‐derived cells must be monitored for genetic instability resulting from long‐term in vitro expansion, PSC‐NK cells—despite having a relatively shorter expansion cycle and lower risk of mutation—may still encounter new genomic safety concerns when strategies involving HLA modulation or gene editing (such as HLA‐E overexpression or functional enhancements) are employed [33]. Third, immunogenicity remains a critical hurdle for PSC‐based therapies due to MHC mismatch. PSC‐NK cells offer innate immunological advantages that support allogeneic use; however, to further enhance graft compatibility, PSC‐NK cells often require additional gene editing (e.g., HLA class I knockout or HLA‐E overexpression). While these modifications may reduce rejection risk, they also introduce new issues related to immune regulation and raise ethical concerns regarding the acceptability of enhanced immune functions [34].

Finally, unlike other PSC‐derived cell types intended for long‐term function (e.g., neurons or pancreatic islet cells), NK cells are inherently short‐lived cytotoxic effectors. Following differentiation, PSC‐NK cells lose their self‐renewal capacity and undergo apoptosis post‐engagement. This transient lifespan necessitates repeated dosing to sustain therapeutic efficacy, increasing treatment burden and placing additional demands on patient adherence. Together, these unique features underscore the need for tailored governance frameworks and translational strategies for PSC‐NK cell therapies [24].

## 3 Ethical and Regulatory Dimensions

**FIGURE 2:**
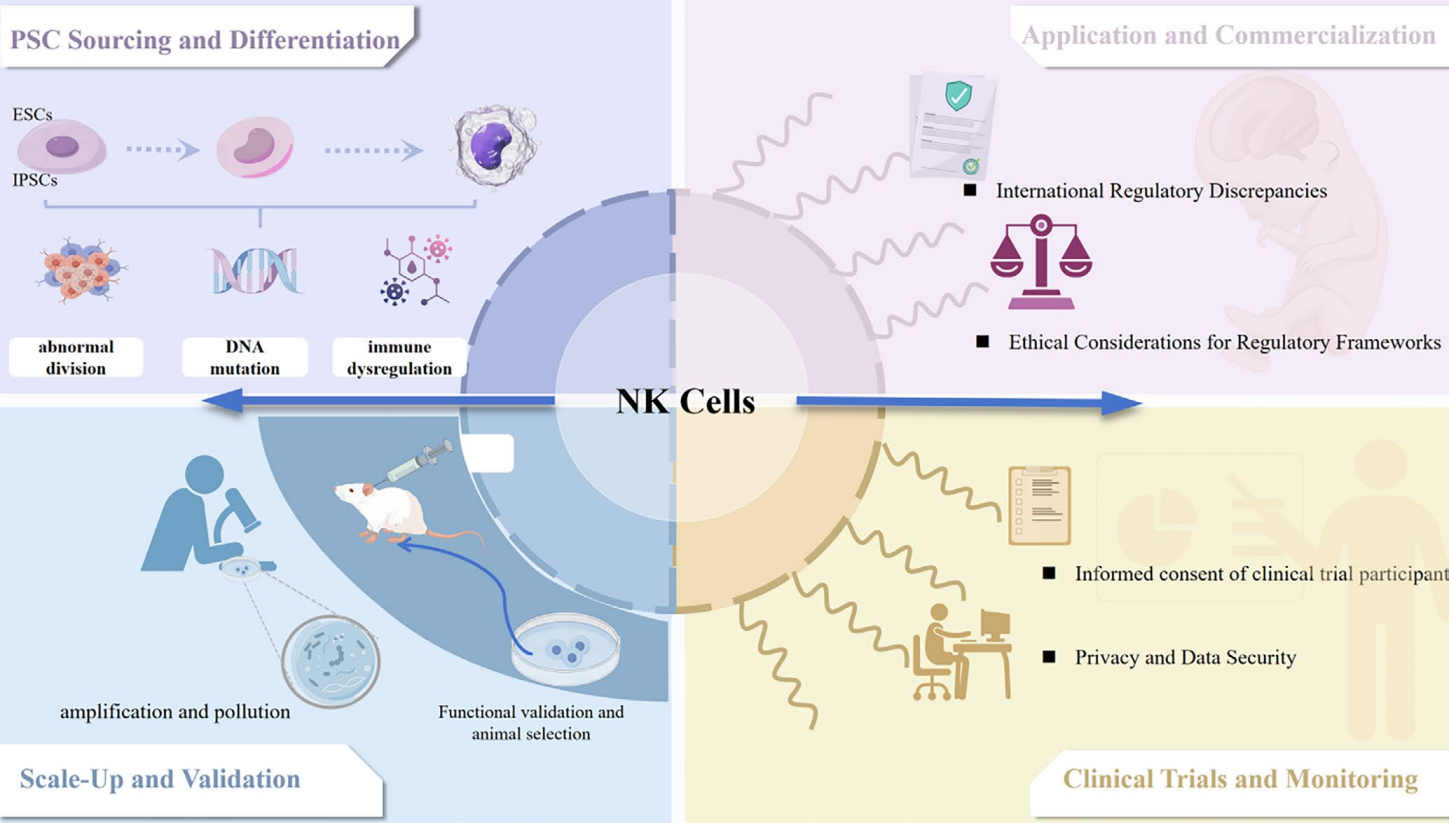
Ethical and safety concerns in PSC‐NK technology.

The clinical translation of PSC‐NK therapies raises ethical and regulatory considerations common to many cell‐based treatments. Their short in vivo lifespan, repeated dosing and immune‐enhancing modifications prompt concerns about patient burden, safety and the scope of cellular intervention (see Figure 2) [35]. As applications expand, issues related to data privacy, consent, and governance also come into focus, underscoring the need to refine oversight frameworks in step with technological advances.

### 3.1 General Ethical and Regulatory Considerations

Within PSC‐based technologies, PSC‐NK cell interventions share a core set of ethical concerns common to other stem cell therapies, spanning the full spectrum from basic research to clinical application. These foundational issues form the ethical baseline for governance in regenerative medicine and include the legitimacy of cell sourcing (i.e., whether PSC lines are derived in accordance with ethical procurement standards and regulatory approvals) [22, 36, 37], adequacy of informed consent from tissue donors at the sourcing stage, and the protection of donor rights. While applicable to PSC‐NK development, these concerns are not unique to this product class.

Additional considerations include material transfer agreements, as well as the traceability and provenance of cell lines across institutional and international boundaries [38]. Further ethical challenges emerge during the clinical translation phase, particularly concerning participant protection, risk‐benefit assessment and the sufficiency of informed consent from trial participants [39]. As PSC‐NK technologies increasingly traverse national borders, they raise additional questions related to global data governance, intellectual property rights and the harmonisation of regulatory frameworks. These dimensions underscore the need for comprehensive, internationally aligned ethical guidance to ensure responsible development and equitable access. However, PSC‐NK cells also give rise to a distinct layer of ethical complexity stemming from their unique immunological function, reliance on extensive genetic modification and application in highly personalised cancer immunotherapies. These concerns are not wholly novel but rather amplified evolutions of the broader ethical dilemmas that characterise PSC research.

### 3.2 Differentiation Purity and Safety Risks

Compared to other PSC‐derived cell types—such as neurons or cardiomyocytes—the differentiation of NK cells is particularly sensitive to cytokine concentrations and activation conditions [40]. This heightened sensitivity has direct implications for the clinical safety of PSC‐NK therapies. Among the foremost concerns is the fidelity of the differentiation process: incomplete or aberrant differentiation may give rise to residual pluripotent cells with distinct biological properties, increasing the risk of tumorigenesis or off‐target immune responses [41]. Such risks not only compromise therapeutic safety but also challenge the ethical justification for clinical use [42].

To mitigate these concerns, safety standards should be grounded in rigorous risk assessments during the differentiation phase, guiding experimental protocols and ensuring the consistent production of functionally mature NK cells [43]. Additionally, trace amounts of residual reagents or growth factors from in vitro culture systems may introduce toxicity risks upon administration. This underscores the need for stringent quality control during the differentiation and manufacturing process to ensure patient safety.

### 3.3 Genomic Stability and Long‐Term Oversight

PSC‐NK cells often require extensive genetic engineering, including CRISPR/Cas9 or TALEN‐mediated modifications, to improve persistence, cytotoxicity and immune evasion in vivo [44]. However, the introduction of multiple genetic edits raises critical concerns regarding genomic integrity. Off‐target effects and unintended genetic alterations may lead to chromosomal instability and elevate the risk of malignant transformation. Variability introduced during reprogramming or differentiation further compounds these risks, potentially compromising both the safety and therapeutic efficacy of the final cell product.

The International Society for Stem Cell Research (ISSCR) guidelines emphasise the necessity of comprehensive genomic stability assessments for genetically modified stem cell products and call for regulatory oversight of highly engineered cell therapies [45]. Beyond professional society guidelines, national regulators have begun addressing genomic stability and genetic modification in PSC‐based products. The FDA requires data on tumorigenicity and chromosomal integrity in IND submissions [46], and China's NMPA emphasises donor screening and genomic analysis in its technical guidelines [47]. While focused on safety, these measures reflect growing attention to ethically salient risks in engineered cell therapies.

Given the irreversible nature of genome editing and the extended proliferative capacity of PSCs, these guidelines underscore the ethical imperative for long‐term monitoring [48]. In the context of iPSC‐based PSC‐NK therapies, vigilant surveillance for genetic and epigenetic abnormalities throughout the differentiation process is essential to mitigate oncogenic potential [49]. In parallel, differentiation‐phase risk assessments should inform safety standards and experimental protocols to minimise clinical hazards.

### 3.4 Manufacturing and Preclinical Biosafety Considerations

Among PSC‐based therapies, PSC‐NK cell products raise distinct ethical concerns stemming from their functional sophistication and genomic engineering. As genetically modified immune effectors, often equipped with CAR constructs or immune checkpoint deletions, PSC‐NKs carry elevated risks during large‐scale manufacturing [50]. Multi‐step differentiation protocols increase the probability of residual undifferentiated PSCs, posing tumorigenic threats. Extended culture and serial passaging further compound risks of chromosomal instability and off‐target genomic events [34]. These challenges necessitate rigorous quality controls, including real‐time molecular surveillance, comprehensive genomic profiling and precise elimination of residual pluripotent cells—amplifying ethical scrutiny around clinical‐grade safety and long‐term monitoring [51].

Unlike many PSC‐derived cell types, which permit extensive in vitro characterisation or limited in vivo validation, PSC‐NK therapies demand robust functional testing in humanised animal models to evaluate immune clearance and tumoricidal activity [52]. This reliance introduces ethical tensions at the interface of genome editing, immune modulation and prolonged animal use. The convergence of human clinical relevance with enhanced animal welfare concerns underscores the need for dedicated ethical review frameworks and heightened procedural transparency to sustain public trust and scientific integrity [53].

### 3.5 Data Privacy and Immunogenomic Ethics

Moreover, privacy protection presents distinct challenges in the clinical deployment of PSC‐NK cell therapies. PSC‐NK cells function as immune effectors, with their cytotoxic activity contingent upon interactions with the host immune system [54], Comprehensive immune profiling is often required prior to treatment to optimise therapeutic efficacy and minimise immunological risks. However, this reliance on detailed immunogenomic data introduces heightened concerns regarding patient privacy, data security, and informed consent, underscoring the need for robust data governance frameworks tailored to the immunotherapy context.

This extensive data collection and processing raises major concerns regarding the integrity of highly sensitive health and genetic information. Potential misuse of or unauthorised access to genetic data may have major ethical and legal repercussions because these data reveal both a patient's health risks and their familial genetic predisposition. Second, applications of PSC‐NK technology often involve collaboration among research institutions, biotechnology companies, and health‐care providers. Ensuring data security during such collaboration across various platforms requires robust data‐sharing agreements and comprehensive cybersecurity measures. This complexity of multiparty collaboration increases the risk of data breaches, necessitating strict privacy protection standards. Third, as an advanced and relatively new therapy, PSC‐NK technology may be subject to evolving regulatory frameworks in different regions. These regional differences in data protection regulations pose certain challenges in ensuring uniform privacy protection for patients participating in clinical trials or receiving treatment across jurisdictions. For example, compliance with the General Data Protection Regulation (GDPR) is required in the European Union, whereas compliance with China's Personal Information Protection Law is mandatory in China [55]. Overall, the unique nature of PSC‐NK technology necessitates the development of tailored privacy protection measures to build trust and achieve a balance between fostering innovation and upholding ethical responsibility.

Addressing the aforementioned challenges requires the establishment of robust cross‐border information‐sharing mechanisms and the development of an internationally coordinated platform that clearly outlines the legality, safety profiles and potential risks associated with PSC‐NK therapy. Unlike traditional stem cell treatments, PSC‐NK therapies, with their technological and ethical implications, require stringent informed consent protocols. These protocols must ensure that patients possess a comprehensive understanding of the scientific foundations, inherent risks, and uncertainties linked to pursuing such treatments. International research and regulatory partnerships must be fostered to mitigate the risks associated with the variability of global regulatory standards. This would promote the alignment of scientific and ethical standards, minimise legal ambiguities and mitigate ethical concerns regarding transnational research and treatment. These measures would contribute to the safe and responsible application of PSC‐NK technology, enhancing public trust and protecting patient welfare on a global scale.

## 4 Conclusions

PSC‐NK technology represents a transformative advancement in cancer immunotherapy, offering a promising alternative for universal, off‐the‐shelf, cell‐based treatment. However, this advancement has also introduced unique ethical, safety and regulatory challenges that must be carefully addressed to foster responsible development. Ensuring clinical safety through measures such as stringent quality control, genomic stability assessments and contamination prevention is essential to ensuring the ethical justification of using PSC‐NK cells. In both preclinical and clinical research, ethical sourcing, culturally sensitive informed consent, and strict adherence to animal welfare principles are essential for maintaining public trust and upholding ethical standards [7]. Overall, the ethical landscape of PSC‐NK research is complicated by challenges such as the demand for global regulatory alignment. Enhanced cross‐border collaboration, transparent data‐sharing practices, and international cooperation are required to unify ethical and scientific standards and thereby address the aforementioned challenges [56]. In the future, advancements in PSC‐NK technology should be guided by a primary ethical framework that prioritises patient safety, data privacy and equitable access. These measures are essential for fostering public confidence, ensuring ethical compliance, and enabling the responsible integration of PSC‐NK therapy into mainstream clinical practice worldwide. Prioritising these considerations can enable the field to advance while upholding scientific integrity and societal values.

## Author Contributions

Y.P., Z.X. and Q.C. conceived and designed the subject. Z.X., J.L. and H.Z. collected data. Q.C., Z.X. and J.L. wrote the paper. Q.C. and X.X. drew the figures and tables. All authors have read and agreed to the published version of the manuscript.

## Ethics Statement

The authors have nothing to report.

## Consent

The authors have nothing to report.

## Conflicts of Interest

The authors declare no conflicts of interest.

## Data Availability

Data sharing not applicable to this article as no datasets were generated or analysed during the current study.
